# Personality Traits, Illness Acceptance, and Psychosocial Outcomes in Patients with Chronic Obstructive Pulmonary Disease: A Cross-Sectional Study

**DOI:** 10.3390/jcm15187243

**Published:** 2026-09-17

**Authors:** Robert Jan Łuczyk, Dorota Weber, Aleksandra Bocian, Marta Łuczyk, Kamil Sikora, Maciej Kaczkowski, Anna Charuta

**Affiliations:** 1 Institute of Health Sciences, Faculty of Medical and Health Sciences, University of Siedlce, 08-110 Siedlce, Poland; anna.charuta@uws.edu.pl; 2 Faculty of Health Sciences, Lublin University of Technology, Projektowa 4, 20-209 Lublin, Poland; dorota.weber@wsei.pl; 3 Department of Internal Medicine and Internal Nursing, Chair of Preventive Nursing, Faculty of Health Sciences, Medical University of Lublin, Ul. Chodźki 7, 20-093 Lublin, Polandkamil.sikora@umlub.edu.pl (K.S.); 4 Long-Term Care Nursing Department, Chair of Preventive Nursing, Faculty of Health Sciences, Medical University of Lublin, Ul. Chodźki 7, 20-093 Lublin, Poland; marta.luczyk@umlub.edu.pl; 5 Department of Health Prophylaxis, Laboratory of Social Nursing, Poznan University of Medical Sciences, 61-701 Poznań, Poland; maciej.kaczkowski@ump.edu.pl

**Keywords:** chronic obstructive pulmonary disease, personality traits, illness acceptance, psychosocial outcomes, quality of life

## Abstract

**Background/Objectives:** Chronic obstructive pulmonary disease (COPD) affects respiratory function, psychological well-being, and quality of life. Personality traits may be associated with adaptation to chronic illness. This study evaluated associations between Five-Factor personality traits, illness acceptance, and selected self-reported psychosocial outcomes in patients with COPD. **Methods:** A cross-sectional study included 120 hospitalized patients who reported a diagnosis of COPD between January 2025 and March 2026. Personality traits were assessed using the NEO Five-Factor Inventory, and illness acceptance using the Acceptance of Illness Scale. Sociodemographic and clinical data were collected with an original questionnaire. **Results:** Participants showed mean sten scores within the average range for neuroticism, extraversion, agreeableness, and conscientiousness, whereas openness was lower than the average range. The mean illness acceptance score was 24.67 ± 2.62, corresponding to moderate acceptance. Higher neuroticism was associated with poorer self-rated adaptation, more frequent dyspnea-related anxiety, and lower self-rated quality of life; neuroticism also differed across categories of daily-functioning limitation (H = 19.909, *p* < 0.001). Higher extraversion was associated with better self-rated adaptation, more favorable self-rated treatment outcomes, and higher self-rated quality of life. Openness correlated negatively with illness acceptance (ρ = −0.338, *p* < 0.001). **Conclusions:** In this sample, personality traits showed unadjusted associations with illness acceptance and selected self-reported psychosocial outcomes. These hypothesis-generating findings should be interpreted cautiously and require confirmation in studies using objective clinical indicators and multivariable analyses.

## 1. Introduction

Chronic obstructive pulmonary disease (COPD) is a common, preventable and treatable disease characterized by persistent, usually progressive airflow limitation associated with chronic inflammatory changes in the airways and lungs, most often caused by exposure to noxious particles or gases, principally tobacco smoke [1]. COPD remains one of the leading causes of morbidity and mortality worldwide, and its prevalence and associated burden are projected to continue rising over the coming decades because of the aging of the population and continued exposure to established risk factors [1,2]. Beyond airflow obstruction, the disease is characterized by a progressive decline in exercise tolerance, recurrent exacerbations and hospitalizations, and a substantial reduction in health-related quality of life, all of which place COPD among the most burdensome chronic non-communicable diseases globally [1,2].

The clinical picture of COPD cannot, however, be reduced to its purely somatic dimension. A growing body of evidence indicates that psychological and psychosocial factors—including anxiety, depressive symptoms, illness perceptions, and personality traits—may be related to how patients experience dyspnea, adhere to treatment, engage in pulmonary rehabilitation, and adapt to life with a chronic respiratory condition [3,4]. Among these factors, personality is of interest because, unlike mood states, it is generally conceptualized as a relatively stable disposition that may be related to the individual’s appraisal of and coping with illness-related stressors throughout the disease course.

The Five-Factor Model (FFM), operationalized through instruments such as the NEO Five-Factor Inventory (NEO-FFI), describes personality along five broad, empirically robust domains: neuroticism, extraversion, openness to experience, agreeableness, and conscientiousness [5,6]. Neuroticism reflects a predisposition to experience negative affect, anxiety, and emotional instability; extraversion reflects sociability, assertiveness, and a tendency toward positive affect; openness to experience reflects intellectual curiosity, imagination, and receptivity to new experiences; agreeableness reflects interpersonal trust, cooperativeness, and altruism; and conscientiousness reflects self-discipline, organization, and goal-directed behavior [6]. These five domains have repeatedly been associated with how individuals perceive, cope with, and adapt to chronic somatic illness [7].

In COPD specifically, personality traits have been associated with disease-specific health status, mental symptoms, and daily functioning. Topp et al. reported associations between neuroticism, agreeableness, conscientiousness, and COPD Assessment Test scores after accounting for concurrent depression and anxiety [3]. Caille et al. found that higher openness to experience was associated with non-response to pulmonary rehabilitation in terms of quality of life [8]. In a Polish sample, Tabała et al. found that patients with obstructive lung disease, including COPD, were characterized by lower conscientiousness and openness to experience and higher trait anxiety and neuroticism than healthy controls, and that these traits differentiated adaptive from maladaptive coping strategies [4]. Illness perceptions and coping strategies, which may be related to personality, have also been associated with quality of life in COPD [9]. Smoking behavior, a central risk factor and frequent comorbid behavior in COPD, has likewise been associated with a distinct personality profile characterized by higher neuroticism and lower conscientiousness, extraversion, and openness relative to non-smokers [10,11].

Illness acceptance—the degree to which a patient comes to terms with the limitations, dependency, and losses imposed by chronic disease—is considered a psychological resource associated with adherence, psychological distress, and quality of life across a range of chronic conditions [12]. Despite the theoretical and clinical relevance of the relationship between personality structure and illness acceptance, this association has not, to our knowledge, been comprehensively examined in patients with COPD using a validated Five-Factor instrument together with a standardized measure of illness acceptance.

The aim of the present study was therefore to describe illness acceptance and selected self-reported psychosocial outcomes among patients with COPD and to examine their associations with Five-Factor personality domains, as measured by the NEO-FFI. The outcomes included illness acceptance, self-rated adaptation to the disease, self-rated quality of life, dyspnea-related anxiety, self-rated treatment outcome, perceived impact of COPD on professional activity and psychological well-being, perceived family support, and smoking status.

## 2. Materials and Methods

### 2.1. Study Design and Setting

A cross-sectional, non-interventional, questionnaire-based study was conducted between January 2025 and March 2026 at the Clinical Department of Pulmonology, Pulmonary Allergology and Internal Medicine with a Sub-Unit of Enhanced Pulmonary Care of University Clinical Hospital No. 4 in Lublin, Poland.

### 2.2. Participants

The study group comprised 120 patients hospitalized in the pulmonology department during the recruitment period who reported a diagnosis of COPD and agreed to participate. The diagnosis was based on the participant’s declaration and was not independently verified against medical records or spirometric results. Patients with acute exacerbations were eligible. Inclusion criteria were a self-reported COPD diagnosis and consent to participate. The only exclusion criterion was inability to provide answers; no separate exclusion criterion based on cognitive impairment or psychiatric status was applied. Patients who could not complete the questionnaire independently but were able to answer were assisted by having the questions read aloud or repeated without suggesting responses. The sample size was determined pragmatically and comprised all eligible patients available during the recruitment period; no formal a priori sample-size calculation was performed. Information on the number of patients assessed for eligibility, invited, or declining participation was not recorded. The variable concerning hospitalization refers to previous hospitalization because of COPD and not to the index hospitalization during which the questionnaire was completed. Participation was voluntary and anonymous; every eligible patient was informed about the aim and anonymous character of the study before completing the questionnaire set, and the introductory section of the survey explained the purpose of the study and instructions for correctly providing answers.

### 2.3. Measures

Three research tools were used:

1. An original sociodemographic and clinical questionnaire consisting of 20 single-choice items concerning the disease entity and coping with it. The questionnaire covered sex, age, marital status, place of residence, education, occupational activity, disease duration, self-rated treatment outcome, hospitalization history, use of oxygen therapy, smoking status and history, family support, and the perceived impact of COPD on daily and professional functioning and psychological well-being. Self-rated adaptation, self-rated treatment outcome, self-rated quality of life, dyspnea-related anxiety, perceived impact on professional activity and psychological well-being, and perceived family support were each assessed with a separate single-choice item.
2. The NEO-Five Factor Inventory (NEO-FFI), developed by Costa and McCrae and used in the Polish adaptation by Zawadzki, Strelau, Szczepaniak, and Śliwińska [6], is a 60-item self-report personality inventory assessing the intensity of the five basic personality domains of the Five-Factor Model—neuroticism, extraversion, openness to experience, agreeableness, and conscientiousness—with 12 items per domain [5,6]. Raw scores were converted to standard ten (sten) scores using the normative tables described in the Polish manual [6]. For descriptive interpretation, sten scores of 1–3 indicate low results, 4 below-average results, 5–6 average results, 7 above-average results, and 8–10 high results. The available study documentation did not specify the use of separate age- or sex-specific normative tables; therefore, We presents raw scores as the primary descriptive metric and sten-based labels are interpreted descriptively.
3. The Acceptance of Illness Scale (AIS), developed by Felton, Revenson, and Hinrichsen and used in the Polish adaptation by Juczyński [12,13], comprises eight statements describing the negative consequences of poor health (limitations imposed by disease, dependency on others, lack of self-sufficiency, and lowered self-esteem). Each item is rated on a 5-point scale, and total scores range from 8 to 40 points; higher scores indicate greater acceptance of illness. For descriptive interpretation, scores of 8–18 indicate low acceptance, 19–29 moderate acceptance, and 30–40 high acceptance [13].

### 2.4. Statistical Analysis

Statistical analyses were performed using the R programming language, version 4.2.2, and the RStudio environment, version 2022.12.0. The level of statistical significance was set at α < 0.05. Inference regarding personality trait levels was based on 95% confidence intervals (CI) for the mean. The Shapiro–Wilk test was used to assess the normality of distributions. Because the study used a pragmatic sample and a broad set of bivariate comparisons, the analyses were treated as exploratory and were limited to unadjusted associations. Associations between personality domains and variables characterizing illness acceptance and the separate self-reported psychosocial outcomes were assessed using the Kruskal–Wallis H test (with Dunn’s post hoc test for pairwise comparisons where applicable), the Mann–Whitney U test, and Gamma and Spearman’s rho correlation coefficients, as appropriate to the measurement scale of the variables. No multivariable models, moderation analysis, or adjustment for multiple comparisons was performed. Accordingly, the reported *p*-values are nominal and should be interpreted cautiously in view of the number of comparisons. Effect sizes are reported as eta-squared (η^2^) for the Kruskal–Wallis test and epsilon-squared (ε^2^) for the Mann–Whitney U test.

## 3. Results

### 3.1. Sociodemographic and Clinical Characteristics of the Study Group

The study group consisted of 120 patients who reported a diagnosis of COPD. Women constituted slightly more than half of the sample, with men forming the smaller group. The largest age group comprised patients over 65 years of age, followed by smaller groups aged 46–55, 56–65, and 36–45 years; approximately one in ten participants was aged 26–35 years, and only two participants were aged 18–25 years. Nearly two-thirds of respondents were married, about one in six was widowed, and smaller groups were unmarried, divorced, or in an informal relationship. Almost half of the participants lived in cities with more than 200,000 inhabitants, about one-quarter lived in rural areas, and smaller groups lived in medium-sized and small towns. With regard to education, the largest group had higher education, followed by secondary education, with smaller groups having vocational or primary education. More than half of the patients were professionally active, while the remainder were retired or receiving a disability pension.

Nearly one-third of patients had been living with COPD for more than 10 years, about one-quarter for 1–5 years, close to one-quarter for 6–10 years, and one-fifth for less than one year. Regarding previous COPD-related hospitalization, three-quarters of patients reported no previous hospitalization because of COPD, whereas one-quarter reported such a history. This variable refers to hospitalization before the index admission during which the questionnaire was completed. Slightly more than half of participants were current smokers, almost one-third were former smokers, and the smallest group had never smoked. Among smokers, over half smoked 10–20 cigarettes daily, about one-third fewer than 10, and the smallest group more than 20 cigarettes daily; the largest group of smokers had smoked for more than 20 years, followed by 5–10 years, 11–20 years, and, least frequently, fewer than 5 years.

### 3.2. Impact of the Disease on Daily Life, Family Support and Psychological Well-Being

Table 1 presents the distributions of self-rated adaptation, self-rated treatment outcome, perceived limitation of daily functioning, perceived impact on professional life, and use of oxygen therapy. Slightly more than half of patients rated their adaptation to COPD as good, about one-third as average, and small groups as poor or very good. Self-rated treatment outcome was rated as good by 49 patients (40.83%), average by 43 (35.83%), very good by 22 (18.33%), and poor by 6 (5.00%). Half of the patients reported that COPD moderately limited their daily functioning, about one-third reported minor limitation, and roughly one-sixth reported considerable limitation. Most patients did not perceive an impact of the disease on their professional life; less than one in ten had ended their professional activity because of COPD, and the smallest group had had to change jobs. Three-quarters of patients did not use oxygen therapy, while one-quarter did.

The majority of participants (86.67%; *n* = 104) reported experiencing support from close relatives, while 13.33% (*n* = 16) reported not experiencing such support (Table 2).

With respect to perceived psychological well-being, dyspnea-related anxiety, and self-rated quality of life (Table 3), nearly half of the patients reported that COPD affected their psychological well-being to a small extent, over one-third reported no effect, and the smallest group reported a considerable effect. The largest group of patients sometimes experienced dyspnea-related anxiety, over one-third rarely experienced it, one in ten experienced it often, and the smallest group never experienced it. Regarding self-rated overall quality of life, over half of patients rated it as good, over one-quarter as average, and smaller groups as poor or very good. These variables were assessed as separate single-item self-reports and are not treated as interchangeable components of one validated psychosocial adaptation scale.

### 3.3. Level of Illness Acceptance

The mean score on the Acceptance of Illness Scale was 24.67 (SD = 2.62; Me = 25; range 18–29) (Table 4). Using the descriptive thresholds reported for the AIS (8–18 = low, 19–29 = moderate, and 30–40 = high acceptance), the majority of patients presented a moderate level of illness acceptance, a smaller group presented a low level, and no patient presented a high level of acceptance.

### 3.4. Personality Profile of the Study Group

Table 5 reports the raw descriptive characteristics of the five NEO-FFI personality domains. For interpretation against the Polish normative scale, mean sten values for neuroticism (4.93), extraversion (5.04), agreeableness (5.49), and conscientiousness (5.28) were within the average sten range (5–6), whereas openness had a lower mean sten value (4.28). Accordingly, openness is described as lower than average in normative terms. Because the available documentation did not specify separate age- or sex-specific normative tables, these labels are descriptive; raw scores remain the primary values reported in Table 5 and used in the subsequent analyses.

#### 3.4.1. Associations Between Personality Domains and Separate Self-Reported Outcomes

Table 6 summarizes the correlations between the five personality domains and illness acceptance, disease duration, self-rated treatment outcome, self-rated adaptation to COPD, dyspnea-related anxiety, and self-rated overall quality of life. These outcomes were analyzed as separate variables rather than as a single validated psychosocial adaptation construct.

Illness acceptance was moderately negatively correlated with openness to experience and weakly positively correlated with agreeableness; no significant association was found with neuroticism, extraversion or conscientiousness. A higher level of illness acceptance was thus associated with higher agreeableness and lower openness to experience.

Disease duration was strongly negatively correlated with openness to experience and weakly negatively correlated with extraversion; no significant associations were found for neuroticism, agreeableness or conscientiousness. A longer duration of COPD was associated with lower openness and lower extraversion.

Self-rated treatment outcome was moderately correlated with neuroticism (negatively) and extraversion (positively), and weakly correlated with openness (negatively) and agreeableness (positively); no significant association was found for conscientiousness. Because the relationship between openness and treatment outcome appeared non-monotonic, Table 7 presents the group distributions for this association. The openness mean was 20.00 in the poor-outcome group, 24.30 in the average-outcome group, 24.57 in the good-outcome group, and 12.18 in the very-good-outcome group. The overall difference was statistically significant (H = 40.081, df = 3, *p* < 0.001, η^2^ = 0.320), with post hoc differences between the very-good group and both the average (*p* < 0.001) and good (*p* < 0.001) groups. This non-monotonic pattern should not be interpreted as evidence that openness determines or predicts treatment outcome; it is an exploratory result based on a single-item self-rated outcome.

Self-rated adaptation to COPD was moderately correlated with extraversion (positively) and neuroticism (negatively); no significant associations were found for openness, agreeableness or conscientiousness. Better self-rated adaptation was thus linked to higher extraversion and lower neuroticism.

Dyspnea-related anxiety was moderately positively correlated with neuroticism and weakly negatively correlated with extraversion; no significant associations were found for openness, agreeableness or conscientiousness. More frequent dyspnea-related anxiety was associated with higher neuroticism and lower extraversion.

Overall quality of life was moderately correlated with neuroticism (negatively) and extraversion (positively); no significant associations were found for openness, agreeableness or conscientiousness. Higher self-rated quality of life was thus linked to lower neuroticism and higher extraversion.

#### 3.4.2. Associations Between Personality Domains and Categorical Self-Reported Variables

Table 8 summarizes nominally statistically significant differences in personality domains across categories of daily-life limitation, impact on professional life, family support, smoking status, and perceived impact on psychological well-being, based on Kruskal–Wallis and Mann–Whitney U tests. All comparisons were unadjusted.

Personality domains differed across categories of daily-functioning limitation. The pattern for neuroticism was non-monotonic: patients reporting considerable limitation had the highest scores, followed by those reporting minor limitation, whereas patients reporting moderate limitation had the lowest scores (H = 19.909, df = 2, *p* < 0.001, η^2^ = 0.153). Extraversion was highest among patients reporting minor limitation and lowest among those reporting considerable limitation (H = 13.569, df = 2, *p* = 0.001, η^2^ = 0.099). Conscientiousness was highest among patients reporting moderate limitation and lowest among those reporting considerable limitation (H = 11.180, df = 2, *p* = 0.004, η^2^ = 0.078). No significant associations were found for openness or agreeableness. Because the categories were based on a single self-report item and the neuroticism pattern was non-monotonic, these findings should be interpreted cautiously.

Regarding the perceived impact of COPD on professional life, group differences were observed for neuroticism, extraversion, agreeableness, and conscientiousness. Patients who had ended their professional activity because of the disease had the highest neuroticism and the lowest extraversion, agreeableness, and conscientiousness scores, whereas patients unaffected in their professional life scored most favorably on these domains (all *p* < 0.05); no significant association was found for openness. These are unadjusted differences in self-reported categories and do not establish directionality.

Perceived family support was associated with lower neuroticism (U = 1429, *p* < 0.001, ε^2^ = 0.179) than no reported support. The present report does not contain inferential test statistics for the other personality domains in this comparison; therefore, no additional conclusions are drawn for them.

With regard to smoking status, non-smokers scored highest on extraversion (H = 17.896, df = 2, *p* < 0.001, η^2^ = 0.136) and openness (H = 19.829, df = 2, *p* < 0.001, η^2^ = 0.152), followed by former smokers, with current smokers scoring lowest on both traits; no significant differences were found for neuroticism, agreeableness, or conscientiousness by smoking status.

Regarding the perceived impact of COPD on psychological well-being, personality domains differed across the three self-reported categories. Patients reporting a considerable impact had the highest neuroticism and lowest extraversion, while those reporting no impact showed the opposite pattern; openness was highest among patients reporting a small impact, and agreeableness was highest among patients reporting no impact. No significant association was found for conscientiousness. These findings describe group differences and should not be interpreted as evidence that personality traits cause psychological impact.

Table 8 includes only statistically significant comparisons from the available results; non-significant comparisons are not reproduced. All results are unadjusted and *p*-values are nominal because no correction for multiple comparisons was applied.

## 4. Discussion

This study examined associations between the Five-Factor personality structure and illness acceptance and selected self-reported psychosocial outcomes in 120 patients who reported COPD, using the NEO-FFI and the Acceptance of Illness Scale. Patients presented mean sten scores within the average normative range for neuroticism, extraversion, agreeableness, and conscientiousness, and a lower mean sten score for openness. The overall AIS score indicated moderate illness acceptance. Because the sample was pragmatic and the analyses were unadjusted, the findings should be regarded as hypothesis-generating.

Neuroticism was the domain most consistently associated with the selected self-reported outcomes: higher neuroticism co-occurred with poorer self-rated adaptation, greater limitation of daily functioning, more frequent dyspnea-related anxiety, lower self-rated quality of life, and greater perceived psychological impact. This pattern is consistent with the findings of Topp et al., who reported relationships between neuroticism, mental symptoms, and the impact of COPD on patients’ daily lives [3]. However, the present cross-sectional data do not establish temporal or causal direction.

Extraversion was associated with more favorable self-rated adaptation, treatment outcomes, and quality of life, as well as with less perceived psychological and professional impact of COPD. The observed pattern may be compatible with the hypothesis that extraverted individuals engage more readily with interpersonal support, but this mechanism was not measured or tested in the present study. These results are broadly consistent with reports linking extraversion to psychological well-being and coping with chronic illness [4,8].

Agreeableness and conscientiousness were associated with selected aspects of functioning. Higher agreeableness was associated with greater illness acceptance, a more favorable self-rated treatment outcome, and a smaller perceived impact of COPD on professional activity. Conscientiousness was associated with group differences in daily-functioning limitation and professional impact; its correlation with self-rated treatment outcome was not statistically significant (γ = 0.142, *p* = 0.061). These findings may be compatible with hypotheses linking these traits to treatment-related behavior and psychosocial functioning, but the relevant mechanisms and independence from clinical covariates were not tested. The literature cited by Tiemensma et al. likewise supports associations among illness perceptions, coping, and quality of life rather than causal conclusions [9].

A notable finding concerned openness to experience: a higher level of this trait was associated with lower illness acceptance and shorter reported disease duration, while its relationship with self-rated treatment outcome was non-monotonic. One possible explanation is that greater openness may be related to more reflective or critical appraisal of health, but this is an untested explanatory hypothesis. Because openness was not significantly associated with self-rated adaptation or quality of life in Table 6, the present finding should not be generalized to overall adaptation. The result is partially consistent with Caille et al., who reported an association between openness and response to pulmonary rehabilitation on quality of life [8].

Perceived family support was associated with lower neuroticism in the present sample. Because support was measured with a single self-report item and the design was cross-sectional, the direction of this relationship cannot be determined. The finding is compatible with, but does not establish, the possibility that personality and perceived support are reciprocally related.

Smoking status was associated with selected personality traits: non-smokers scored higher on extraversion and openness to experience than current smokers. This pattern could reflect differences in health-related behavior, personality, or their combination, but the cross-sectional design does not permit conclusions about directionality. The results are broadly consistent with studies in the literature reporting associations between smoking status and personality profiles [10,11].

Taken together, the present findings suggest that personality traits are related to selected self-reported indicators of illness acceptance and psychosocial functioning in patients who reported COPD. The analyses do not show whether personality precedes these outcomes, whether the associations are independent of clinical factors, or whether unmeasured factors account for part of the observed pattern. A high neuroticism score may be compatible with less adaptive coping, but coping strategies were not directly measured in this study. Personality-related information could nevertheless be considered in future research on individualized psychosocial assessment, provided that it is combined with objective clinical indicators and validated outcome measures.

### Strengths and Limitations

This study has several limitations that should be considered when interpreting the results. First, the cross-sectional design precludes causal inference and does not establish the direction of the observed associations. Second, the pragmatic sample was drawn from a single pulmonology department, which may limit generalizability and introduces a risk of selection bias; recruitment-flow data were not recorded. The COPD diagnosis was based on participant declaration and was not independently verified against medical records or spirometry, and the specific reason for the index hospitalization was not systematically recorded, although patients with acute exacerbations were eligible. Third, all clinical and psychosocial variables other than the AIS were based on self-report, and several important outcomes—self-rated adaptation, treatment outcome, quality of life, dyspnea-related anxiety, family support, professional impact, and psychological impact—were measured with single items rather than dedicated validated scales. Fourth, objective indicators of disease severity, including FEV1, GOLD (Global Initiative for Chronic Obstructive Lung Disease) stage, exacerbation history, and comorbidity burden, were unavailable or not captured. Fifth, potentially relevant available covariates, including age, sex, disease duration, smoking status, occupational status, and oxygen therapy, were not included in multivariable models; depressive symptoms, anxiety, cognitive functioning, and comorbidity burden were not assessed. Sixth, no formal a priori sample-size calculation was performed, no correction for multiple comparisons was applied, and the available report contains only significant categorical comparisons. Finally, internal-consistency estimates for the NEO-FFI domains and AIS were not calculated in the present sample. The main strengths are the use of Polish adaptations of two established instruments and the assessment of several separate self-reported indicators. Future studies should use independently verified diagnoses, objective disease-severity measures, validated multi-item outcomes, transparent recruitment-flow reporting, and adequately powered longitudinal or multivariable designs.

## 5. Conclusions

1. In this pragmatic, single-center cross-sectional sample of patients who reported COPD, the mean AIS score indicated moderate illness acceptance. Mean sten scores were within the average normative range for neuroticism, extraversion, agreeableness, and conscientiousness, whereas openness had a lower mean sten score.
2. Higher neuroticism was associated with poorer self-rated adaptation, more frequent dyspnea-related anxiety, lower self-rated quality of life, and greater perceived limitations and psychological impact. Higher extraversion was associated with more favorable self-rated adaptation, treatment outcome, and quality of life.
3. Agreeableness and conscientiousness were associated with selected categorical indicators of functioning, while openness was associated with lower illness acceptance and shorter disease duration. The openness–treatment outcome pattern was non-monotonic and should be regarded as exploratory.
4. These findings are unadjusted associations and do not establish causal relationships, moderation, or independent effects. Given the self-reported diagnosis, single-item outcomes, absence of objective disease-severity indicators, lack of multivariable adjustment, and absence of multiple-testing correction, the results should be considered hypothesis-generating and require confirmation.

## Figures and Tables

**Table 1:** Self-rated adaptation, treatment outcome, daily functioning, professional activity, and use of oxygen therapy (*n* = 120).

Domain	Category	*n*	%
Self-rated treatment outcome	Poor	6	5.00
	Average	43	35.83
	Good	49	40.83
	Very good	22	18.33
Self-rated adaptation to COPD	Poor	8	6.67
	Average	41	34.17
	Good	68	56.67
	Very good	3	2.50
Limitation of daily functioning	Minor	39	32.50
	Moderate	60	50.00
	Considerable	21	17.50
Impact on professional life	None	103	85.83
	Yes, had to change job	7	5.83
	Yes, ended professional activity	10	8.33
Use of oxygen therapy	No	91	75.83
	Yes	29	24.17

**Table 2:** Experience of family support among patients with COPD (*n* = 120).

Family Support	*n*	%
No	16	13.33
Yes	104	86.67

**Table 3:** Self-rated psychological well-being and quality of life among patients with COPD (*n* = 120).

Domain	Category	*n*	%
Perceived impact of COPD on psychological well-being	No	44	36.67
	Yes, to a small extent	57	47.50
	Yes, considerably	19	15.83
Dyspnea-related anxiety	Never	12	10.00
	Rarely	42	35.00
	Sometimes	52	43.33
	Often	14	11.67
Self-rated overall quality of life	Poor	9	7.50
	Average	34	28.33
	Good	73	60.83
	Very good	4	3.33

*n*—number of observations; %—percentage.

**Table 4:** Level of illness acceptance (AIS) in the study group (*n* = 120).

Variable	M	SD	Me	Min	Max	Skewness	Kurtosis	Shapiro–Wilk W	*p*
AIS total score	24.67	2.62	25	18	29	−0.57	−0.368	0.931	<0.001

M—mean; Me—median; SD—standard deviation.

**Table 5:** Descriptive characteristics of NEO-FFI personality domains (raw scores; normative sten interpretation described in the text; *n* = 120).

Personality Domain	M	SD	Me	Min	Max
Neuroticism	20.79	7.10	20.0	4	44
Extraversion	25.02	6.57	26.0	8	47
Openness to experience	21.98	7.08	22.0	9	34
Agreeableness	30.18	5.53	30.5	15	41
Conscientiousness	30.98	6.80	32.0	13	48

M—mean; Me—median; SD—standard deviation; sten—standard ten score. Table 5 reports raw scores; normative sten interpretation is provided in the text. All five distributions departed significantly from normality (Shapiro–Wilk, *p* < 0.05), which justified the use of non-parametric statistics in subsequent analyses.

**Table 6:** Correlations between NEO-FFI personality domains and illness acceptance, disease duration, treatment outcome, adaptation, dyspnea-related anxiety and quality of life.

Variable	Coefficient	Neuroticism	Extraversion	Openness	Agreeableness	Conscientiousness
Illness acceptance (AIS)	ρ	−0.045 (*p* = 0.628)	0.111 (*p* = 0.227)	**−0.338 (*p* < 0.001)**	**0.237 (*p* = 0.009)**	−0.030 (*p* = 0.742)
Disease duration	γ	−0.071 (*p* = 0.403)	**−0.190 (*p* = 0.032)**	**−0.508 (*p* < 0.001)**	−0.045 (*p* = 0.581)	−0.099 (*p* = 0.172)
Self-rated treatment outcome	γ	**−0.350 (*p* < 0.001)**	**0.336 (*p* < 0.001)**	**−0.292 (*p* = 0.005)**	**0.201 (*p* = 0.005)**	0.142 (*p* = 0.061)
Self-rated adaptation to COPD	γ	**−0.301 (*p* = 0.003)**	**0.308 (*p* = 0.005)**	−0.191 (*p* = 0.059)	0.125 (*p* = 0.230)	0.047 (*p* = 0.669)
Dyspnea-related anxiety	γ	**0.482 (*p* < 0.001)**	**−0.292 (*p* < 0.01)**	0.085 (*p* = 0.284)	−0.104 (*p* = 0.296)	−0.131 (*p* = 0.168)
Overall quality of life	γ	**−0.492 (*p* < 0.001)**	**0.411 (*p* < 0.001)**	−0.128 (*p* = 0.217)	0.109 (*p* = 0.308)	0.130 (*p* = 0.230)

ρ—Spearman’s rank correlation coefficient; γ—Gamma correlation coefficient. Statistically significant coefficients (*p* < 0.05) are shown in bold. All *p*-values are nominal, and no adjustment for multiple comparisons was applied.

**Table 7:** Openness to experience by self-rated COPD treatment outcome—exploratory Kruskal–Wallis H-test comparison.

Treatment Outcome	*n*	M	SD	Me	Mr
Poor	6	20.00	0.00	20	40.00
Average	43	24.30	3.43	25	71.27
Good	49	24.57	6.38	26	71.39
Very good	22	12.18	6.23	10	20.80

H = 40.081; df = 3; *p* < 0.001; η^2^ = 0.320. M—mean; SD—standard deviation; Me—median; Mr—mean rank.

**Table 8:** Summary of nominally statistically significant associations between NEO-FFI personality domains and categorical clinical or psychosocial variables (Kruskal–Wallis H and Mann–Whitney U tests).

Clinical/Psychosocial Variable	Personality Domain	Test Statistic	df	*p*	Effect Size
Limitation of daily functioning	Neuroticism	H = 19.909	2	<0.001	η^2^ = 0.153
	Extraversion	H = 13.569	2	0.001	η^2^ = 0.099
	Conscientiousness	H = 11.180	2	0.004	η^2^ = 0.078
Impact on professional life	Neuroticism	H = 13.115	2	0.001	η^2^ = 0.095
	Extraversion	H = 6.729	2	0.035	η^2^ = 0.040
	Agreeableness	H = 8.867	2	0.012	η^2^ = 0.059
	Conscientiousness	H = 7.463	2	0.024	η^2^ = 0.047
Family support (yes vs. no)	Neuroticism	U = 1429	—	<0.001	ε^2^ = 0.179
Smoking status	Extraversion	H = 17.896	2	<0.001	η^2^ = 0.136
	Openness	H = 19.829	2	<0.001	η^2^ = 0.152
Impact on psychological well-being	Neuroticism	H = 23.403	2	<0.001	η^2^ = 0.183
	Extraversion	H = 34.189	2	<0.001	η^2^ = 0.275
	Openness	H = 18.167	2	<0.001	η^2^ = 0.138
	Agreeableness	H = 15.070	2	0.001	η^2^ = 0.112

## Data Availability

The data presented in this study are available upon request from the corresponding author.

## References

[1] Global Initiative for Chronic Obstructive Lung Disease (GOLD) (2024). Global Strategy for the Diagnosis, Management, and Prevention of Chronic Obstructive Pulmonary Disease: 2025 Report.

[2] Meng K., Chen X., Chen Z., Xu J. (2025). Burden of chronic obstructive pulmonary disease in adults aged 70 years and older, 1990–2021: Findings from the Global Burden of Disease Study 2021. PLoS ONE.

[3] Topp M., Vestbo J., Mortensen E.L. (2016). Personality traits and mental symptoms are associated with impact of chronic obstructive pulmonary disease on patients’ daily life. COPD.

[4] Tabała K., Wrzesińska M., Stecz P., Kocur J. (2016). Personality traits, level of anxiety and styles of coping with stress in people with asthma and chronic obstructive pulmonary disease—A comparative analysis. Psychiatr. Pol..

[5] Costa P.T., McCrae R.R. (1992). Revised NEO Personality Inventory (NEO-PI-R) and NEO Five-Factor Inventory (NEO-FFI): Professional Manual.

[6] Zawadzki B., Strelau J., Szczepaniak P., Śliwińska M. (1998). Inwentarz Osobowości NEO-FFI Costy i McCrae: Adaptacja Polska.

[7] Adam L.C., Halmschlager N., Zimmermann J., Franke C., Knaevelsrud C., Kerber A. (2026). Associations between maladaptive personality traits and physical health: Results of a cohort study with a large representative sample. J. Psychosom. Res..

[8] Caille P., Alexandre F., Molinier V., Heraud N. (2021). The role of personality traits in inpatient pulmonary rehabilitation response in patients with chronic obstructive pulmonary disease. Respir. Med..

[9] Tiemensma J., Gaab E., Voorhaar M., Asijee G., Kaptein A.A. (2016). Illness perceptions and coping determine quality of life in COPD patients. Int. J. Chronic Obstr. Pulm. Dis..

[10] Terracciano A., Costa P.T. (2004). Smoking and the Five-Factor Model of personality. Addiction.

[11] Stephan Y., Sutin A.R., Luchetti M., Caille P., Terracciano A. (2019). Cigarette smoking and personality change across adulthood: Findings from five longitudinal samples. J. Res. Pers..

[12] Felton B.J., Revenson T.A., Hinrichsen G.A. (1984). Stress and coping in the explanation of psychological adjustment among chronically ill adults. Soc. Sci. Med..

[13] Juczyński Z. (2011). Narzędzia Pomiaru w Promocji i Psychologii Zdrowia.

